# Conclusions: Capturing, Sustaining, Spreading and Researching Change

**DOI:** 10.1007/978-3-030-62662-4_9

**Published:** 2020-11-24

**Authors:** Louise Ackers, Gavin Ackers-Johnson, Joanne Welsh, Daniel Kibombo, Samuel Opio

**Affiliations:** 6 Global Social Justice, University of Salford, Salford, UK; 7 University of Salford, Salford, UK; 8 Infectious Disease Institute, Kampala, Uganda; 9 Pharmaceutical Society of Uganda, Kampala, Uganda

**Keywords:** Sustainability, Public–private partnership, Policy transfer, Snagging

## Abstract

This chapter summarises the overall impacts of the Maternal Sepsis Intervention reflecting on the processes of capturing, sustaining and spreading best practice in antimicrobial stewardship. It argues that sustainability is achievable when an intervention reduces hospital costs and, in such cases, the responsibility or sustainability rests with LMIC institutions. Key barriers to sustainability include supply chain weaknesses and human resource limitations. The chapter recommends the use of Public–Private Partnerships to help to overcome these barriers.

Dyar et al. (2017) review the use of the term ‘antimicrobial stewardship’ and conclude that there has been an overemphasis on conceptualisations focused on ‘individual prescriptions’ and insufficient emphasis on the societal implications of antimicrobial use. Furthermore, and of particular relevance to the MSI project, there has been insufficient translation of the concepts of ‘responsible use’ into context and time-specific actions. The authors conclude that AMS is not so much a concept as it is a tool to assess whether organisations are identifying actions to improve responsible use in the specific context within which they are functioning. This idea fits very well with the action-research approach used in our intervention and the results arising from that.

The final chapter includes three sections. The first section ‘Capturing Change’ summarises the effectiveness of the intervention identifying key outcomes and the drivers that have contributed to these. The second section ‘Sustaining Change’ addresses the issue of sustainability of those change processes, and the third section ‘Spreading Change’ discusses the opportunity to translate and transfer aspects of the approach within the hospital, throughout the country and to other international contexts. We draw the chapter to a close reflecting on the benefits of the ethnographic approach and the attention to contextual detail and co-researching that this has afforded.

## Capturing Change

Although we were acutely aware of the importance of Infection Prevention and Control (IPC), the emphasis in the CwPAMS call on more complex concepts of AMR and AMS distracted us somewhat from the foundational importance of IPC. Poor attention to IPC in the hospital clearly contributed to high levels of protracted and expensive hospital-acquired infection creating more and more unpleasant work for health workers. IPC improvement has proved to be a key driver of behaviour change. Health workers have viewed the changes as an immediate investment in their own health and safety, improving their ability to work effectively in a safe, supported environment. As such IPC has not just improved the ‘opportunity’ component of behaviour change, it has engaged directly in health worker motivation. Attempting to address AMS without full attention to IPC would, we believe, have failed to capture the imagination and commitment of ward staff. Capability (or lack of knowledge or skill) did not emerge as a key factor in behaviour change, and where knowledge mobilisation has taken place, it has occurred primarily through mentoring and role modelling with some attention to specifics (like when hand gel should be used in relation to washing or what concentration of JIK should be used to clean mattresses). Nurses and midwives have emerged as the natural leaders in these areas playing a key role in shaping organisational culture and orienting rotating doctors and students. The financial costs of implementing major improvements in the IPC infrastructure have been minimal in comparison with the savings achieved through shorter hospital stays, reductions in re-admissions and use of theatre for secondary wound closures.

Change has come about over time through a myriad of small changes and attention to the limitations or unintended consequences of these. The process has generated active and positive dialogue and collegiality. Once the ‘obvious’ issues around hand hygiene and equipment sterility had been resolved, the management of attendants emerged as a key and stubborn weakness in IPC systems. Whilst progress is being made and has been accelerated in the COVID-19 environment, further work needs to be done in this area throughout the hospital and at national level. The impact of stock-outs on the ability to maintain basic hygiene on the wards is another structural issue requiring intervention at the level of national and institutional supply chains. We return to this issue below.

The IPC work focused attention on wounds and wound care on the wards. On one level, this was very much an IPC issue; improving IPC reduces the risk of wounds becoming infected on the wards in the first place. Managing those that do become infected (or arrive infected) reduces the risk of sepsis and the need for secondary closure. Cleaning and dressing wounds emerged as a key, if unforeseen, dimension of infection control; it also provides the opportunity for swabbing and, with that, for complex multi-disciplinary ‘ huddling’. At this point, the nurses and midwives hand the baton to the laboratory and microbiology results create a unique opportunity for multi-disciplinary dialogue grounded in scientific results. Once these results are available, so the opportunity for clinical pharmacy emerges with pharmacists engaging actively with intern doctors to support evidence-based, ‘rational’ prescribing. The overwhelmingly positive impacts of these processes on patient outcomes, the environment on the ward and the satisfaction at being able to save lives and shorten hospital stays feeds back into the loop creating an active momentum for change. This in turn generated an appetite for knowledge, to breach disciplinary boundaries, share a new language of communication and experience mutual respect.

The findings evidence significant and impactful behaviour change on the wards with genuine multi-disciplinary team working contributing to changes in prescribing behaviour and AMS. Wound care and laboratory testing lie at the heart of these changes centre staging nurses, midwives and laboratory scientists in stewardship processes. The results of the microbiology testing then provide a platform for genuine multi-disciplinarity and, specifically, the first opportunity for clinical pharmacy engagement. This is true both at the level of rational (evidence-based) prescribing for individual patients and in improving the evidence base behind empirical prescribing (through an understanding of resistance patterns). If access to a full range of antibiotics were available, this platform of behaviour change would transform antimicrobial use patterns, reduce the over- and inappropriate use of antimicrobials, improve patient outcomes and deliver significant cost-benefits.

The changes described above are essential to achieving ‘responsible use’ in a Ugandan public hospital; but they are not sufficient. Cox et al. make the important point that, *‘delayed or no access to antibiotics kills more people than antibiotic resistant bacteria. … AMS is not only about reducing inappropriate use, but also assuring access to effective treatment’* (2017: 813). The changes create the opportunity for active pharmacy engagement in multi-disciplinary decision-making. This achievement could have a major impact on antibiotic use as post-natal and gynae wards consume by far the largest volume of antibiotics at the hospital. The cost-effectiveness of this intervention underlines the sustainability potential and the immediate opportunity for scale-up across the hospital as a whole and to other public health facilities in Uganda and beyond.

Chapter 10.1007/978-3-030-62662-4_6 has elaborated the complexity and opacity of the supply chain system in a public hospital setting in Uganda. In the absence of effective supplies not only will these cost savings elude facilities; the patients involved will fail to thrive and the motivation of health workers to apply the skills and knowledge they have demonstrated will inevitably decline. We have explained in detail the complex dynamics of supply chain management. Understanding and piecing together these processes have required painstaking ethnographic research to unpick major errors in record-keeping and interpret the trends observed. In the first instance, the very centralised system creates huge dependency on the functionality of National Medical Stores and the adequacy of Ministry budgets. Centralisation may be seen as necessary in systems so damaged by corruption but where this undermines flexibility and responsiveness and generates extended and predictable stock-outs, the systems put in place to improve antimicrobial stewardship will, inevitably, fail.

The lack of supervision of rotating intern doctors by their senior colleagues restricts the effectiveness of the approach and the osmotic potential of continuous multi-disciplinary engagement. Nurse and pharmacy leaders have filled the knowledge gap through orientation and mentoring of intern doctors demonstrating the potential of task shifting in practice. Further improvement to prevent surgical site infection requires attention in the operating theatre as women undergo caesarean sections or laparotomies and in complex secondary wound closures. The deployment of a UK doctor in theatre had a very positive effect often substituting for the absence of senior doctors. Ultimately, this is unsustainable, and measures need to be taken to ensure the constant presence of senior doctors in obstetric theatre and in a supervisory capacity in public hospitals.

The change mechanism deployed in the MSI is grounded in a particular approach to knowledge mobilisation and behaviour change. Building on extensive experience of project operationalisation, the team was acutely aware of the limited efficacy of fly-in-fly-out formal training programmes organised by foreign teams and lubricated by opportunities to leave the ward or receive per diems. We have established the working principle of co-presence with colleagues working side by side on the wards in more democratic and genuinely bilateral^1^ mentoring relationships. Where Ugandan health workers have articulated a desire to gain new knowledge, the project has mobilised to provide that. In some cases, this has involved short sessions led by UK experts—such as the interpretation of laboratory results, for example. In others, the whole group of pharmacy interns presented a workshop on AMR to the hospital. Horizontal knowledge mobilisation between different disciplinary groups is a feature of this process rather than a comparison of protocols or practices in high- and low-income countries.

Co-presence is not only a principle to be applied to health worker training; it also forms the basis of ethical and equitable approaches to co-researching.

## Sustaining Change

Sustainability has become a key concept in overseas development interventions. As with most things, sustainability needs to be understood in context; it is also highly contested. Sustainable Development is defined in the 1987 Brundtland Report (United Nations 1987) as: ‘Development that meets the needs of the present without compromising the ability of future generations to meet their own needs’. Thwink.org^2^ provides a very simple definition that resonates with the objectives of the MSI, describing sustainability as, ‘the ability to continue a defined behaviour indefinitely’. The powerful emphasis on sustainability in international development has arisen largely due to increasing recognition of the ineffectiveness of aid and, in some cases, the damaging externality effects associated with aid dependency (Moyo 2010). We have noted the limited evidence of the impact of many foreign-supported training initiatives including the many that have failed to mobilise knowledge from theory into practice and impact behaviour. The MSI has demonstrated genuine and sustained behaviour change in Infection Prevention Control and rational prescribing. But can this behaviour be sustained?

Implicit in many discussions of sustainability is the notion of an ‘exit-strategy’ to guard against funding dependency. Whilst we are very aware of the damaging effects of foreign aid dependency, we would argue that exit is not a zero-sum game. Continuity of relationships and partnership has been the hallmark of K4C’s engagement in Uganda. It is the quality of transitions from a phase of more intense engagement to a more routine maintenance that is of importance here. Many aid interventions are characteristically brief resulting in rapidly designed interventions followed by immediate withdrawal. The CwPAMS funding is typical in that regard; the funding period was extremely short with no lead time and discrete measurable and attributable outcomes expected within 15 months. The co-creation and co-working model has ensured high levels of local engagement and genuine team working both within the hospital and between project partners (in Uganda and the UK).

## Sustainability and Human Resource Augmentation

There can be no doubt that individual capability (knowledge and skill) to improve AMS exists amongst all cadres. In many areas, this existed prior to intervention particularly in relation to a fundamental understanding of infection prevention control and wound management. Health workers also referred to gaining new, more specialist, knowledge and implementation skills which would ensure continuity and were clear that the work would continue after project funding ends. The feedback from ward midwives evidences their recognition of the new knowledge that has come with the project and their belief that they are now able to maintain the behaviour change on the ward. However, they all expressed concerns about the impact that staff shortages will have on their ability to deliver a quality service. In some respects, the project has increased the complexity of tasks on the ward; midwives are now more actively engaged in new tasks such as swabbing; managing the process of sending samples to the laboratory and receiving them (which used to be the role of the doctors); and then pulling the teams together to act on lab results. There is also an increased burden on the staff and the in-charge to manage patients and visitors and ensure all new students and interns are effectively oriented in the culture and practices of the ward. There is an important task-shifting component here as the in-charge and pharmacist are effectively fulfilling the role of the senior doctor in orienting intern doctors. On the other hand, measures to enable midwives to work more effectively, reducing frequent visits to surgical theatre for sterilising equipment and gauze and physically visiting the lab for test results, have reduced demands on staff. In reality, the K4C staffing team settled into a routine of having one person on the ward every day of the week (mornings only at weekends and no nights). The costs of this additional and perhaps dedicated midwife could be considered money well spent by the hospital if they are keen to maintain the model and the cost savings it has brought.

The success of the MSI is not contingent on the skills or knowledge of short-stay foreign volunteers, or on foreign income sources. Critically, the intervention has been recognised by the hospital as cost saving:

When sepsis is managed then the resources used to manage these patients reduce drastically. By reducing long stays which brings about savings. You have contributed to the hospital budget with real term savings (Hospital Administrator).

The findings evidence the overwhelming success of the approach in terms of reducing infection risks; managing infections more effectively; improving prescribing behaviour; and reducing maternal morbidity and mortality. In the process, the evidence of reductions in patient stays, use of operating theatres and readmissions represent significant cost savings for the hospital. Asked about the sustainability of the model at an IPC meeting, Professor Ackers replied:

Everything we have done here is completely sustainable. We have not put in big fancy investments or one-off trainings. We have saved the hospital considerable funds; the responsibility for sustainability now rests with the hospital. I don’t think having one extra member of staff is too difficult for the hospital to maintain. We have not brought in some big thing that we are now taking away.

Indeed, to push this point further, an extension of the MSI approach to other wards in the hospital would result in further cost savings. In this context, where an intervention has improved outcomes whilst also reducing costs, the responsibility for sustainability rests with the hospital and health system to put systems in place. This would imply some investments and delegation of national autonomy—particularly in employing more nurses/midwives in place of the K4C staff and more pharmacists to support clinical pharmacy on other wards. These measures are within the hospital plans and jurisdiction and fully actionable. More detailed exposure of costs may lubricate the politics of internal decision-making. A discussion about the mechanics of undertaking meaningful and accurate cost-benefit analysis to identify the financial impact of the project has taken place with hospital management. Certainly, accurate assessment of costs would facilitate evidence-based human, and overall, resource management. And failure to make these decisions and augment staffing to protect the model, post-project, will undoubtedly result in cost escalation as patient stays begin to increase. The Health Partnership team are committed to supporting this process. There can be no doubt that managing the volume of patients on the wards presents serious challenges. One option for the hospital management to consider is the use of creative financial management to reinvest a portion of the cost savings achieved through the intervention model to employ additional staff.

The impact of damaged referral systems and the ability of referring hospitals and health centres to bump patients on to referral hospitals with impunity and take no responsibility for the mortalities and morbidities they contribute to require the engagement of the Ministry of Health.

## Sustainability and Access to Supplies

Access to supplies, in relation to IPC, wound management and antimicrobials, has proved an ongoing concern. Although substantial improvements have been made, these are sub-optimal and realising the full impact of the approach depends on improving access to supplies. Aid dependency is not simply about the size of financial commitments in relation to local income; it is also about the mode of engagement. The concept of ‘conditionalities’ has been deployed in an attempt to reduce no-strings-attached ‘giving’ or ‘donations’ which undermine sustainability. In general, simply ‘donating’ consumables can never be sustainable and it can and does encourage new forms of corruption. It is for this reason that K4C as a charity does not routinely engage in these forms of support.

We are acutely aware that our ability to make even very small payments can save lives. During the MSI, we made one such payment (of £30) to purchase amikacin when we knew that, in the absence of that payment, the mother would almost certainly die. The problem here is not just about costs; it is structural. Amikacin is not currently available through National Medical Stores, and as the hospital budget for drugs is held at NMS, the solution requires more structural changes which we hope to support through active advocacy work (see below). K4C also gave the ward two bottles of JIK when supplies ran out. This cost £6 but safeguarded the improvements in basic IPC on the ward. The problem here was caused by NMS stock-outs. K4C has also been supplying hand gel to the ward for the duration of the intervention. The decision to engage in what looks like unsustainable investments was made cautiously; K4C has the ability to manufacture hand gel locally and the costs of doing so are quite low.^3^ The decision was made to provide it given the total lack of supplies and the fundamental importance of hand hygiene to the achievement of behaviour change.

Concerned about the ability to sustain hand gel production in the previous project (funded by the Tropical Health and Education Trust), K4C proposed a Public–Private Partnership agreement with FPRRH. K4C generates income to sustain its own activities between project funding, through its student placement programme (Ahmed et al. 2017). In most hosting facilities, K4C makes investments, based on Fair Trade Principles, including the provision of staff and infrastructure, etc., on a pro-rata basis in recognition of support for the placements. FPRRH had insisted on cash payments (of £150 per student placement) rather than the in-kind investments deployed in other health facilities. K4C had proposed that at least some of this payment could be made into a PPP account, especially as K4C provides supervision for students through its own staff co-working on the wards. This income could then be utilised to underwrite the costs of IPC. At the time, this proposal was rejected by the hospital director. The proposal was raised again during the MSI and the start of the COVID-19 pandemic. At this point, K4C was keen to support the hospital to put IPC systems in place across all its wards. A Public–Private Partnership agreement has now been signed generating a more sustainable and integrated mechanism for supply chain augmentation with an emphasis on IPC and antimicrobials. This provides a unique opportunity for foreign organisations to cooperate on a co-decision and co-funding basis, guided by the hospital’s Medicines Therapeutic Committee and supported by a reputable not-for-profit supplier, Joint Medical Stores. The objective will be to move away from dependency-generating donations to a more integrated approach with the agility to respond to local needs.

O’Neills review of AMR (2016) identified a number of ‘guiding principles’ for implementation. These included an emphasis on cost-effectiveness and the identification of ‘market failures affecting resource allocation, regulation and price mechanisms’. Most of the solutions advocated in his review engage at international level; the proposed PPP mechanism we identify operates at micro-institutional level^4^ and we hope will become a scalable model. This represents an important opportunity to improve sustainability at the local level. Detailed evaluation of this mechanism will contribute to a wider dissemination strategy that will support advocacy work at national and international levels.

## Spreading Change

The MSI has demonstrated the potential for change and the efficiencies associated with this. We hope that publication of this evidence will stimulate discussion at national level amongst all key stakeholders and generate a momentum for change.

## Policy Transfer Opportunities Within the Hospital

The most immediate opportunity to scale up and apply the MSI approach lies with the hospital itself. The Infection Prevention Control (IPC) Committee has already played an important role in exposing key players in the hospital to the changes in practice introduced on the post-natal and gynae wards. It is interesting to note that some of the concerns expressed on PNG wards about the fluidity of intern rotations have created an interest in and appetite for change as health workers who have spent time on PNG subsequently move to other wards. From a governance perspective, further work needs to be done to fully establish and institutionalise the hospital Medicines Therapeutic Committee (MTC). This process has been complex and circuitous. The pharmacy team acknowledged the central importance of the MTC in policy implementation processes. Whilst FPRRH is one of only very few RRHs in Uganda to have functionalised the Rx medicines management system, the pharmacy team expressed concern that, in the absence of an MTC, the full impact of this could not be realised:

Before we didn’t have the people to operate the Rx system. It helps us know about the supply chain and which drugs are about to expire. In theory we should be doing reports and submitting them to the MTC, but the committee is still not functioning.

There is also concern that some of the measures developed are not fully recognised by hospital doctors because they have not been validated through an established MTC:

The ‘Antibiotic Selection Tool’ we had designed has been removed from the wall; we think it is because it has not gone through the MTC.

This illustrates the importance of the MTC to the validation and endorsement of policy and medicines management. Whilst considerable efforts have been made on the post-natal and gynae ward to block the prescription of high-end antibiotics without lab tests, extending this policy across the whole hospital requires MTC endorsement. The project co-lead and Secretary General of the Pharmaceutical Society of Uganda expressed a similar concern about the role of the MTC as a platform for the utilisation and dissemination of data and the institutionalisation of the model used on post-natal and gynae wards. We propose a stepwise cautious incremental approach to the scaling up in other wards commencing in either surgical ward or paediatrics. The COVID-19 pandemic has thrown a light on supply chain efficacy and the impact of weak supply chains on global and national inequalities. Although the poorest in societies will suffer disproportionately, the tentacles of antimicrobial resistance, as with all global pandemics, will reverberate across the globe.

There is a growing concern embodied in evolving research governance that ethical research demands attention to dissemination. This includes active engagement with stakeholders and policymakers and aligns with the principles of partnership commitment. This also extends to a commitment to accessibility as Harding suggests:

Research should not be elite [..] it would have to be accessible – physically and intellectually to anyone interested. It would be humble and acknowledge that each new “truth’ is partial; that is incomplete as well as culture-bound’ (1991: 300).

Open access publishing plays a key role in this process; it also demands attention to the use of language to ensure that all actors in multi-disciplinary audiences are able to make sense of research whilst also not over-simplifying key messages.

## Researching Change: Ethnography and ‘ Snagging’

Providing the evidence base to inform and facilitate behaviour or systems change processes is notoriously complex even when researchers are working in their own country and institutions. Add to that the complexity of power relations and the dynamics of organisational culture in very different national contexts, and it is easy to understand how interventions in global health can fail. Co-working and co-researching have combined in this study through a form of action-oriented ethnography. Ethnography, as a profoundly inductive and intuitive approach has enabled a whole series of small ‘snags’^5^ or ‘knots’ to be identified and responded to with a level of agility that has enabled the intervention to move forward in a reflexive process. And this has made people feel that the intervention and research have been attentive; that their views have been listened to, respected, and responded to. Challenging AMR demands attention to detail, to the minutiae and mundane features of health workers’ everyday lives.

The next section reflects on some of the prescriptions and assumptions that shape the global and national response to AMR and are echoed in the CwPAMS ‘positioning’ that framed the funding programme. We outlined the Ugandan National Action Plan—itself an implementation device of the Global Action Plan. Certainly, all five areas resonate with our intervention findings as key priorities. Whilst it is valuable to distinguish the areas, they are essentially indivisible and interrelated. With the exception of surveillance, where scientific methodologies can help shed light on the mechanisms, transmission patterns and origins of bacteria and resistance, we would argue that any intervention must span all 5 areas. Cox et al.’s review (2017) identifies the lack of evidence on AMS derived from highly contextualised interventions. The focus on maternal sepsis in our work will, we hope, represent a response to that call with context here not referring simply to work taking place in LMICs but to work closely engaged with health system priorities and a patent local need. Embedding AMR/AMS work in this way creates a powerful resonance and tangibility about the impact of what are, in many ways, quite intangible concepts. The CwPAMS call, with funding of £1.3 m, represents a ‘drop in the ocean’ in the wider AMR funding landscape both in terms of UK funding to LMICS and that of other foreign actors. On that basis, we can understand the desire for focus. There is much discussion about partnerships in intervention delivery but less attention to partnerships between funding bodies. In many respects, the growing tendency for funding bodies to come together to support ‘real-world’ problems and combine interventions with greater attention to evaluation is to be welcomed. Disciplinary and professional boundaries have generated myopic impressions of the world. However, there are some unintended consequences of these partnerships that demand careful and honest reflection. Funding is fundamentally a political process. Chapter 10.1007/978-3-030-62662-4_2 referred to the dominance of certain paradigms in global health and the heavily prescribed nature of many funding calls. Inevitably, these lead to a deductive approach shaped by powerful normative assumptions. Shiffman discusses the role that power plays in shaping global health agendas. At one level, power shapes health priority-setting and this is clearly seen in instruments such as the Sustainable Development Goals. Reflecting on the role that the Lancet has played in global health agenda-setting, Shiffman poses the question: ‘Why do some individuals become recognised as global health experts’? (2014: 297). We have noted the dominance of medical and pharmaceuticalisation paradigms that have come to shape discourses around AMR and global health more generally. Our work has challenged these paradigms both from a methodological perspective (what constitutes knowledge in this context) and from a task-shifting (clinical) perspective (which cadres have the greatest potential to support antimicrobial stewardship in LMICs?).

The team have worked with THET for many years and we share many of the values embedded in the principles of health partnership working. We feel these align closely with an action-research and fundamentally inductive approach and support the concept of locally grounded, highly contextualised, case studies. They also support the need for more democratic knowledge mobilisation mechanisms formed around continuous mentoring and co-working relationships. This concurs with the ethos of the Sustainable Development Goal 17 (global partnerships). The Department of Health and Social Care is a very different organisation with its primary objectives, necessarily (and rightly in our view) focused on the needs of the UK in what are very challenging times. AMR is posing a genuine and immediate threat to the UK economy and the health and well-being of its citizens. It goes without saying that any funding committed to LMICs must at least demonstrate ‘mutual interest’. Our own previous work funded by Health Education England (Ackers et al 2017) reported on the benefits to the NHS from professional volunteering identifying gains in key areas such as leadership, communication and managing in resource-constrained environments. The Commonwealth Pharmacist Association, as a body representing the interests of pharmacy as a profession inevitably and necessarily, has vested interests too. The CPA describes its role in terms of: ‘Empowering pharmacists to improve health and wellbeing throughout the Commonwealth’.^6^ Once again, this is an important and much needed perspective. There is substantial under-investment in pharmacy in Uganda, so much so that it is impossible for pharmacists to utilise their clinical pharmacy knowledge and skills in public facilities. Our ongoing engagement with FPRRH made us very aware that this was the case prior to project inception and the impacts of this emerged over the project, as did the potential for change when employing just one more pharmacist to support the work. What became profoundly clear though was that engagement of clinical pharmacy in the ways that the CPA planned required the engagement of laboratory scientists. Without laboratory, testing the potential for clinical pharmacy on the wards is severely compromised. And the opportunity to take samples for laboratory testing rested on the respect for and active co-working with those cadres that are continually present on the wards. In the Ugandan context, this means midwives and nurses.

The funding bodies necessarily had their own, political, objectives arising from the need to demonstrate impact on an increasingly cynical and cash-limited environment. These objectives combined in complex and ‘clunky’ ways to make project specification, management and evidence-generation extremely difficult. The final component of the AMR NAP (and GAP) refers to research. Good research needs space to define and change priorities, and research excellence is increasingly associated with ‘impact’. Evaluation is research and should be distinguished from accountability mechanisms. It is interesting to note that little, if any, of the log-frame ‘evidence’ we were required to provide at regular intervals to the funding bodies has been used in this book, and little, if any, of the key evidence presented in the book could be squeezed into the log-frames.

The experience of managing this project has been characterised by ongoing creative tension with the funding bodies and managing agents bound by (and required to police) highly prescriptive, inflexible and deductive log-frame systems. Somekh describes ‘episodes of substantial friction’ as ‘the starting point for deeper collaboration […] co-labouring with partners involves encountering many ‘knots’ associated with discomfort, difficulty and frustration’ (2006: 23). Friction, she argues, reflects the deep seriousness that partners attach to their work. We hope that the outcomes achieved will encourage a debate in Overseas Development Assistance work to allow projects and researchers to breathe, innovate, show the humility to acknowledge intervention failure and prepare to be surprised.

## Footnotes

1 The team have been allocated Commonwealth Fellowships to support 5 Ugandan health workers to come to the UK for 3 months to develop their learning around tissue viability and wound management.

2 https://www.thwink.org/sustain/glossary/Sustainability.htm.

3 The costs of providing hand gel to the PNG during the project came to around £100.

4 The MOH, Uganda has recommended to development of PPP as part of its Health Strategic Health Policy. This is echoed in the National Medicines Policy (2015: 30) that promotes PPP in the pharmaceutical sector.

5 The term ‘snag’ is often used in building contracts to refer to ‘a problem or difficulty that stops or slows the progress of something’.

6 https://commonwealthpharmacy.org.
